# Benefits of Enhanced Phase-Locking for Binaural Coding of Amplitude-Modulated Sounds

**DOI:** 10.1177/23312165261421708

**Published:** 2026-02-11

**Authors:** Go Ashida

**Affiliations:** 1 Department of Neuroscience, 11233 Carl von Ossietzky Universität Oldenburg, Oldenburg, Germany; 2 Cluster of Excellence “Hearing4all”, Carl von Ossietzky Universität Oldenburg, Oldenburg, Germany

**Keywords:** sound localization, binaural hearing, auditory brainstem, temporal coding, cochlear nucleus, superior olivary complex, computational modeling

## Abstract

The neural processing of interaural time and level differences (ITDs/ILDs) underlies binaural sound localization. Neurons of the mammalian lateral superior olive (LSO) are sensitive to ILDs and envelope-ITDs of acoustic stimuli. Bushy cells in the anteroventral cochlear nucleus convey relevant information from auditory nerve (AN) fibers to the LSO. More specifically, spherical bushy cells (SBCs) send ipsilateral excitatory inputs, while globular bushy cells (GBCs) project to the contralateral medial nucleus of the trapezoid body that provides inhibitory inputs to the LSO. Previous studies in vivo reported an enhancement of phase-locking in bushy cells compared to AN. This enhancement has been hypothesized to benefit temporal coding in binaural neurons, but its actual contribution in LSO remains unclear. Here we investigate this question by computational modeling of binaural circuity incorporating the AN, SBC/GBC, and LSO stages. Both bushy cell models were calibrated to replicate known physiological responses, including the representative peristimulus time histograms for high-frequency tones and enhanced phase-locking to low-frequency envelopes. We then simulated the binaural tuning of LSO with and without the bushy cell stage. The synaptic inputs to the LSO model were adjusted so that the simulated ILD-tuning remains unaltered between the input configurations. By adding the bushy cell stage, the simulated binaural response of LSO became more sharply tuned for envelope-ITDs. Furthermore, the envelope-ITD sensitivity was extended up to around 600 Hz, matching previously observed physiological limits. These results provide computational evidence for the functional benefit of having bushy cells in the binaural sound localization circuit.

## Introduction

**Figure 1.**
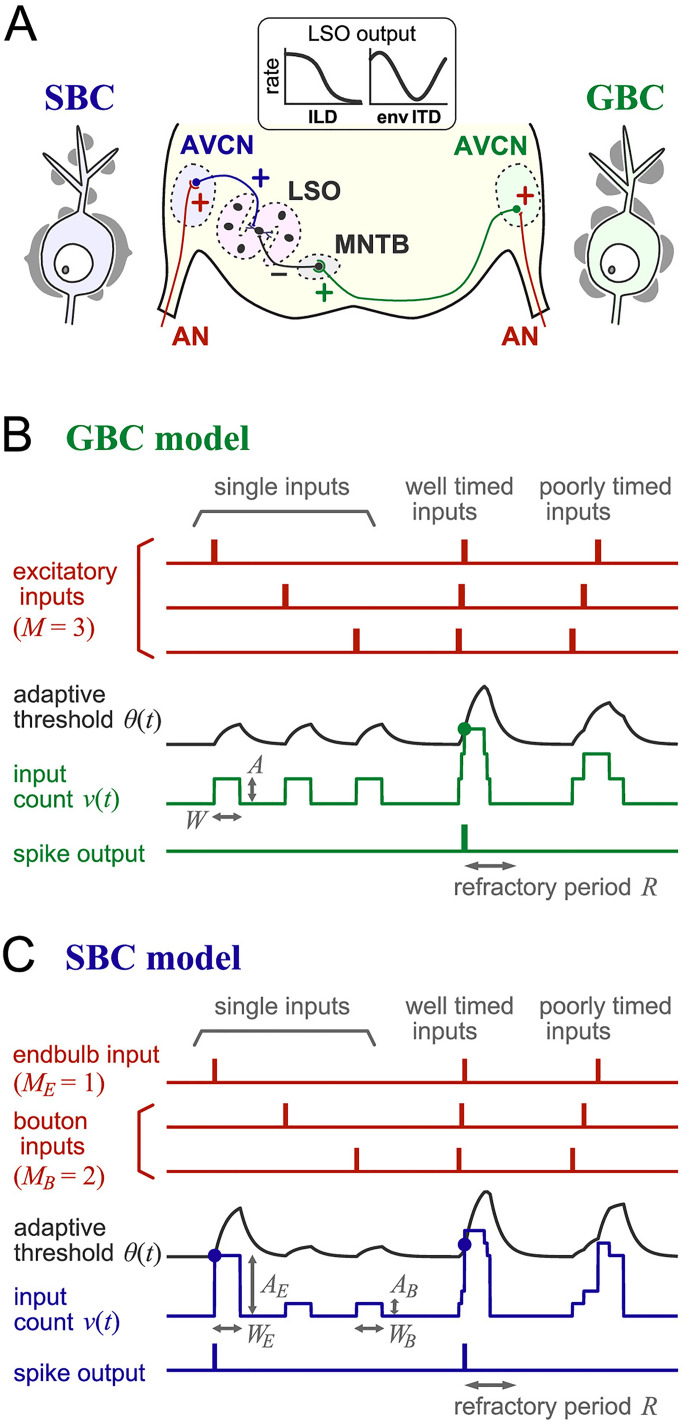
**Modeled binaural circuit. (A)** Schematic drawing of the LSO circuit and synaptic projections on bushy cells. A spherical bushy cell (SBC) in the AVCN receives a small number of large endbulb synapses on the soma along with a larger number of small bouton synapses. A globular bushy cell (GBC) in the AVCN receives an intermediate number of medium-sized modified endbulb synapses. The plus signs in the circuit indicate excitatory synaptic connections, while the minus sign between the MNTB and LSO shows inhibition. The output spike rate of an LSO neuron varies with ILDs and envelope ITDs. AN: auditory nerve; AVCN: anteroventral cochlear nucleus; LSO: lateral superior olive; MNTB: medial nucleus of the trapezoid body. Adapted from Fig. 1A of Ashida et al. (2017). **(B)** Schematic operation of the GBC model. The model neuron receives *M* excitatory inputs from simulated AN fibers (*M* = 3 in this example). Each presynaptic AN spike (small vertical red bars) is converted into a rectangular postsynaptic response (input count) of a duration *W* and height *A*. The threshold *θ*(*t*) adaptively develops according to the summed input count *v*(t). Convergence of well-timed inputs leads the input count to reach the threshold (green dot) to generate an output spike (small vertical green bar). After each spike output, the model is in the refractory period of a duration *R*. For poorly timed inputs, the adaptive threshold increases more rapidly than the input count and no spikes are produced. **(C)** Schematic operation of the SBC model. The example AN spikes (red) are the same as for the GBC model. The SBC model, however, receives *M_E_* endbulb and *M_B_* bouton inputs from AN fibers (*M_E_* = 1 and *M_B_* = 2 in this example). The amplitude *A_E_* of the endbulb input is barely suprathreshold, exceeding the threshold (first blue dot on the left) if there is no (or little) prior increase in the adaptive threshold. The amplitude *A_B_* of the bouton inputs is small, but well-synchronized occurrence of endbulb and bouton inputs leads to a crossing of the threshold to produce a spike (second blue dot). Spike generation is rejected for poorly synchronized inputs, because the increase of the threshold due to prior inputs effectively moves the endbulb input from the suprathreshold regime to subthreshold.

Binaural sound localization in humans and animals relies on the neural processing of relevant acoustic cues, such as interaural time and level differences (ITDs/ILDs: Grothe et al., 2010; Yin et al., 2019). The lateral superior olive (LSO) in the mammalian auditory brainstem is one of the earliest locations where information from the two ears converges (Friauf et al., 2019; Tollin, 2003). Sustained-firing neurons in the LSO are sensitive to ILDs of binaurally presented sounds (Boudreau & Tsuchitani, 1968; Irvine et al., 2001; Tsai et al., 2010). In addition, LSO neurons respond differentially to ITDs of clicks and amplitude-modulated (AM) sounds (Batra et al., 1997; Irvine et al., 2001; Joris & Yin, 1995, 1998). In response to binaurally presented AM sounds, the firing rate of an LSO neuron changes periodically with the envelope time disparity, termed the “envelope ITD.” Acoustic stimulation on one ear excites the ipsilateral LSO and inhibits the contralateral LSO (Figure 1A). Excitatory synaptic inputs to LSO neurons are mediated by spherical bushy cells (SBCs) in the ipsilateral anteroventral cochlear nucleus (AVCN). Inhibitory inputs to LSO are provided by neurons in the medial nucleus of the trapezoid body (MNTB) that receive strong excitatory projections from globular bushy cells (GBCs) in the contralateral AVCN (Cant & Benson, 2003). For the processing of acoustic cues transferred bilaterally via SBCs and GBCs, LSO neurons act as “anticoincidence detectors” that vary the spiking output by comparing their excitatory and inhibitory synaptic inputs on a submillisecond time scale (Ashida et al., 2016; Beiderbeck et al., 2018; Tollin & Yin, 2005). Both types of bushy cells (BCs) have short membrane time constants mediated by low-voltage activated channels (Cao et al., 2007; Rothman & Manis, 2003), and receive rapid synaptic inputs from auditory nerve (AN) fibers (Cao & Oertel, 2010; Gardner et al., 1999; Isaacson & Walmsley, 1996). These specializations are required for the generation of action potentials with high temporal fidelity to convey the timing information of sounds towards higher stages including LSO (Manis et al., 2012; Wu & Kaczmarek, 2021). While sharing similar morphologies of round-shaped somata and relatively compact dendrites (Osen, 1969), the synaptic structures on SBCs and GBCs are distinct from each other. SBCs receive a small number of large synaptic terminals called endbulbs of Held (Ryugo & Sento, 1991; Sento & Ryugo, 1989), as well as a larger number of small bouton synapses (Gómez-Nieto & Rubio, 2009; Ryugo & Sento, 1991). AN synapses on GBCs, called modified endbulbs, are generally smaller than the endbulb terminals on SBCs (Ostapoff & Morest, 1991; Ryugo & Rouiller, 1988; Spirou et al., 2005; 2023).

The spiking activity of AN fibers rises preferentially at a certain phase of periodic acoustic stimuli, such as low-frequency pure tones and amplitude-modulated (AM) sounds with a low-frequency envelope (reviewed in Heil & Peterson, 2015; Joris et al., 2004). The synchrony between the sound phase and neuronal spike times, called phase-locking, plays a fundamental role in ITD coding of tones and AM sounds (Grothe et al., 2010; Yin et al., 2019). A number of physiological recording studies have reported an enhancement of phase-locking in cochlear nucleus neurons including SBCs and GBCs (Frisina et al., 1990; Joris et al., 1994; Joris & Yin, 1998; Paolini et al., 2001; Recio-Spinoso, 2012; Rhode & Greenberg, 1994a; Typlt et al., 2012; Wei et al., 2017, 2023), suggesting that sharpening of temporal tuning is one of the most crucial physiological functions of BCs. Analyses of spike trains indicated that increased temporal precision from AN to BC might facilitate coincidence detection in the downstream neuron (Joris et al., 2006; Louage et al., 2006). Furthermore, a signal detection approach applied to noise-driven activity of ANs and BCs suggested a considerable improvement of envelope ITD sensitivity at high frequencies (van der Heijden et al., 2011). It is still unknown, however, how the enhancement of phase-locking in BCs affect the binaural coding in LSO. To address this question, we developed simple BC models based on relevant anatomical and physiological findings. In the following text, we present these models and verify the biological plausibility of their responses. We then connect the BC models to the binaural LSO model to investigate their roles in ITD processing of AM sounds. We also explore the hypothetical scenario in which BCs are bypassed in the binaural LSO circuit.

## Methods

The model framework of this study has three stages: AN fibers, BCs, and an LSO neuron. The AN stage (including the auditory periphery) receives acoustic inputs and generates a sequence of action potentials. The modeled BCs are driven by AN outputs and send their spike outputs to the binaural LSO stage (Figure 1A). The structure of each stage is described in the following subsections.

### Auditory Nerve Model

Acoustic stimuli were fed into the 2018 version of an established auditory periphery model (Bruce et al., 2018) to simulate the spiking activity of AN fibers. The model was developed on the basis of physiological data from animal studies (cats and rodents), and offers an option to fit human data. We used the “cat” option of the model, as the physiological data we aimed to replicate were primarily taken in cats. Since cat BCs receive inputs mostly from high-spontaneous-rate (SR) fibers (Liberman, 1991; but see the Discussion section), we set the SR of the model AN fibers to 70 (spikes/s). The refractory periods of the model were fixed at the midpoint of its default parameter ranges (absolute: 0.45 ms, relative: 0.5125 ms).

### Globular Bushy Cell Model

To simulate the activity of GBCs, we used the “adaptive coincidence counting” model, which was confirmed to reproduce fundamental response patterns of GBCs (Ashida et al., 2019). A presynaptic AN spike induces a rectangular postsynaptic response (Figure 1B), which represents the spike count in a sliding time window of duration *W*. The total synaptic input *v*(*t*) (which we also refer to as the summed input count) is a linear sum of all postsynaptic response. The adaptive spiking threshold of the model is a sum of static and dynamic components: *θ*(*t*) = *θ*_S_ + *θ*_D_(*t*). The static component is time-invariant and is fixed to one: *θ*_S_ = 1. The amplitude *A* of each rectangular postsynaptic response is normalized to this static threshold. The dynamic component *θ*_D_(*t*) of the threshold varies according to the summed input count *v*(t) with a time constant *T*. Namely, *T*(d*θ*_D_/d*t*) = -*θ*_D_(*t*) + *S*·*v*(*t*), with the parameter *S* being the strength of adaptation. This adaptive threshold model roughly approximates various biological mechanisms, such as voltage-dependent activation and inactivation of ion channels and inhibitory inputs from other neurons in the cochlear nuclei, which underlie the temporally precise spiking activity of BCs (see the discussions of Fontaine et al. (2013) and Ashida et al. (2019)). A spike output is recorded at the time point when the summed input count *v*(*t*) reaches or exceeds the threshold *θ*(*t*). Immediately after each spike, the model enters a refractory period of duration *R*, in which no further spikes are triggered. Because of the adaptative threshold, the model generates an output spike only with sufficiently synchronized inputs by rejecting poorly synchronized inputs (Figure 1B). The model parameters are summarized in Table 1.

**Table 1.** Parameters of the BC Models.

Parameter	GBC Model	SBC Model
Input amplitude	*A* = 0.40	*A*_E_ = 1.05 (endbulb), * A*_B_ = 0.21 (bouton)
Input duration (ms)	*W* = 0.5	*W*_E_ = 0.5 (endbulb), *W*_B_ = 0.5 (bouton)
Refractory period (ms)	*R* = 1.2	*R* = 0.7
Adaptation strength	*S* = 0.9	*S* = 0.9
Adaptation time constant (ms)	*T* = 0.3	*T* = 0.3

### Spherical Bushy Cell Model

Both GBCs and SBCs share similar membrane properties (e.g., Cao et al., 2007; Jing et al., 2025). Accordingly, previous modeling studies usually did not distinguish their membrane potential dynamics, assuming that their distinct spiking responses observed in vivo originate from the different innervation patterns of AN fibers (e.g., Rothman et al., 1993; Rothman & Young, 1996). We adopted this assumption and modified the input structure of GBC model to simulate the activity of SBCs, while keeping the general model concept and the threshold adaptation mechanism unaltered. Reflecting the anatomical findings in SBCs (reviewed in the Introduction), our SBC model receives two types of inputs, mimicking the postsynaptic potential amplitudes for large endbulb synapses and small bouton synapses. The amplitude of an endbulb input *A*_E_ is slightly higher than the static threshold, while the amplitude of a bouton input *A*_B_ is subthreshold (Figure 1C). Under acoustic stimulation, for which input fibers are all active, the impact of endbulb input can shift from supra- to subthreshold due to adaptation, reflecting the activity-dependent variation of responses in SBCs (Keine & Rübsamen, 2015; Kuenzel et al., 2011). We chose the same duration parameter for the endbulb (*W*_E_) and bouton (*W*_B_) inputs as for the modified-endbulb synaptic input of the GBC model (see the Discussion section for the limitation of this assumption). The SBC model has a shorter refractory period than the GBC model, allowing strong endbulb inputs to generate spikes with a shorter dead time. We note that increasing the refractory period generally leads to a shift of the simulated PSTH shape from primary-like (=SBC-type) to primary-like-with-notch (=GBC-type). The model parameters are summarized in Table 1.

### LSO Neuron Model

The excitatory-inhibitory interaction in LSO was simulated with a (non-adapting) “coincidence counting” model that was confirmed to replicate the monaural and binaural responses of sustained-firing LSO neurons (Ashida et al., 2016, 2017). The model parameters (coincidence threshold, coincidence window, inhibition strength, inhibition window, and refractory period) were unchanged from these previous studies.

### Connecting the AN Stage to the BC Stage

Based on the narrow frequency tuning of BCs comparable to AN (Rhode and Smith, 1986), we assumed that both types of BC models receive inputs from AN fibers sharing the same characteristic frequency (CF). Tonotopic variations of cellular and synaptic properties were not considered for the BC models (see the Discussion section). Anatomical reconstructions revealed that a GBC receives on average ∼20 AN terminals in cats (Ostapoff & Morest, 1991; Spirou et al., 2005). We therefore assumed *M* = 20 AN inputs for each GBC model, as adopted previously (Ashida et al., 2019). The number of endbulb terminals on an SBC is typically two in cats (Liberman, 1991; Ryugo & Sento, 1991; see comments in Joris & Smith, 2008). The number of small bouton inputs is largely unknown (Gómez-Nieto & Rubio, 2009). In the present study, we assumed *M*_E_ = 2 endbulb inputs and *M*_B_ = 18 bouton inputs for each SBC model. A previous study used 32 bouton inputs as the default for a gerbil SBC model (Koert & Kuenzel, 2021). Our preliminary simulations agreed with their findings that the simulated SBC responses do not depend on the specific number of bouton inputs if it is larger than 15–20.

### Connection to the LSO Stage

In order to investigate the roles of BCs in the sound localization circuit, we compared three different input configurations to LSO: (1) the “AN-only” configuration in which AN fibers from both sides directly project to the LSO neuron without BCs; (2) the “AN + GBC” configuration in which inhibitory inputs driven by contralateral sounds are provided via the GBC model, while the ipsilateral AN fibers directly send excitatory projections to LSO; and (3) the “SBC + GBC” configuration in which inputs from both sides are provided via the BC models. We adjusted the number of inputs within the experimentally observed ranges (Gjoni et al., 2018; Sanes, 1990), so that the simulated ILD-tuning curves (especially the maximum and minimum spike rates) resembled each other for these three configurations, also matching previous physiological measurements (see Results). In the mammalian LSO circuit, neuron in MNTB receive excitatory inputs from GBCs and send inhibitory inputs to LSO (Figure 1A). The calyx of Held synapse between GBC and MNTB provide very secure one-to-one connection (Englitz et al., 2009), and the activity of an MNTB neuron closely inherits the activity of a GBC (Borst & Soria van Hoeve, 2012). We therefore assumed the MNTB to be a reliable sign inverter without failure, and directly used the GBC outputs as inhibitory inputs to LSO.

### Sound Stimuli (Pure Tones)

Pure-tones were used to test the physiological plausibility of BC model responses. The frequency was varied between 150 and 9,000 Hz. The default sound intensity was 70 dB SPL, unless otherwise noted. The CF of the model matched the frequency of the tone, because we only considered on-CF responses. Each stimulus presentation contained a tone burst of 25 ms with a linear rise/fall time of 3.9 ms (Joris et al., 1994). We repeated 2,000 stimulus presentations to calculate the output measures of the model (see below). For the analysis of sustained responses, we used spikes occurring within a time window of 10–25 ms after the stimulus onset (Joris et al., 1994).

### Sound Stimuli (AM Tones)

Sinusoidal amplitude-modulated tones were used for further testing of the BC responses, and for simulating the binaural responses of LSO neurons. The carrier frequency of the sound, as well as the CF of the models, were fixed to 7,000 Hz. A low-frequency sinusoidal envelope (100–700 Hz, 100% modulation depth) was applied to the carrier wave. The default sound intensity was 35 dB SPL, unless otherwise noted. The selected default level was lower than that for pure tones, as phase-locking declines for high intensity AM sounds (see Results). The duration of the sound was 420 ms, and the spike response for the first 20 ms was discarded from the analysis of sustained responses. For BC responses, we repeated 3,000 trials to calculate the output spikes of the models. For LSO responses, we used 100 repetitions. We note that the total number of repetitions required for the BC stage is generally ∼30 times larger than that for the LSO stage, because the LSO neuron receives converging BC outputs.

### Phase-Locking

The degree of phase-locking of spike trains was quantified by vector strength (VS: Goldberg & Brown, 1969; Kessler et al., 2021), which is defined as: $\mathrm{VS}=(1/N)\left|\overset{N}{\underset{k=1}{\sum }}\;\exp\;(2\pi ift_{k})\right|$ , where *N* is the number of spikes, *i* is the imaginary unit, *f* is the reference frequency, *t_k_* is the timing of the *k*-th spike. For pure-tone stimulation, *f* is the frequency of the tone. For AM-tone stimulation, *f* is the frequency of the envelope.

### Measures of Binaural Tuning

For the simulation of ILD-tuning, we used unmodulated 7,000 Hz tones, with the ipsilateral level fixed to 35 dB SPL and the contralateral level being varied. The ILD is defined as the contralateral level minus the ipsilateral level (Irvine et al., 2001; Joris & Yin, 1995). For the simulation of envelope-ITD-tuning, we fixed the sound level to 35 dB SPL and varied the relative phase of the envelope of the binaural stimuli within the range of ±180 degrees. A positive phase difference indicate that the sound wave arrives earlier at the contralateral ear than at the ipsilateral ear (Irvine et al., 2001; Joris & Yin, 1995). We did not explicitly incorporate synaptic and/or axonal delays to the connections of the model stages, because the measured conduction time from AN to LSO is comparable for both sides (Joris & Yin, 1998) and our focus of binaural coding is solely on the sustained response without onset transients (see the Discussion section).

### Numerical Simulation and Code Availability

All model components were simulated in Matlab with a time step of 0.01 ms (100 kHz sampling rate). The model code and test scripts are available online at https://github.com/pinkbox-models/SBC2025.

## Results

The present study aims to assess how the known enhancement of synchrony in BCs affects the coding of sound-localization cues in LSO. To this end, we have developed a model of SBCs by expanding the synaptic input structure of the existing GBC model (see Methods). Here we first explore the biological plausibility of the simulated BC model responses by comparing them with relevant physiological data recorded in cats. Data from other mammals are used for further reference. We then incorporate the BC stages into the modeled LSO circuit, and examine their roles in binaural coding of AM sounds.

### Pure-Tone Responses of BC Models

**Figure 2.**
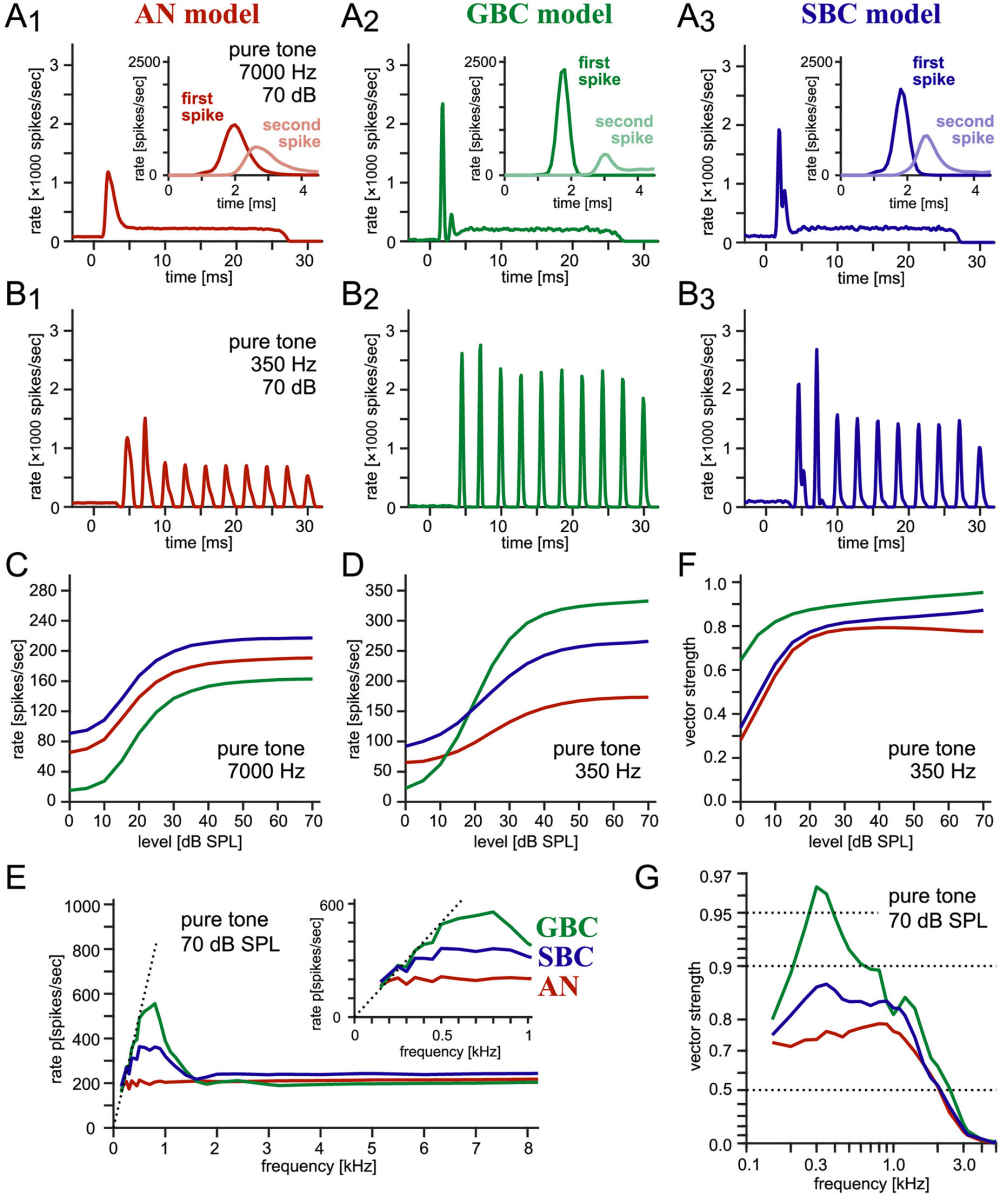
**Simulated responses for pure tones. (A_1_–A_3_)** Peristimulus time histograms (PSTHs) of the AN model (red, A_1_), GBC model (green, A_1_), and SBC model (blue, A_3_), driven by 7,000 Hz tone at 70 dB SPL. **(Inset of A_1_–A_3_)** Histograms of first (dark color) and second (light color) spike latencies. **(B_1_–B_3_)** PSTHs of the AN (B_1_), GBC (B_1_), and SBC (B_3_) models, driven by 350 Hz tone at 70 dB SPL. **(C)** Rate-level curves for the AN, GBC, and SBC models at 7,000 Hz. **(D)** Rate-level curves for the AN, GBC, and SBC models at 350 Hz. **(E)** Rate-frequency curves at 70 dB SPL. The inset shows the zoomed image for 0–1,000 Hz. **(F)** VS-level curves for the three models at 350 Hz. **(G)** VS-frequency curves at 70 dB SPL. The vertical axis is on the inverted logarithmic scale of (1-VS) as in previous studies (Johnson, 1980; Joris et al., 1994).

In response to high-frequency pure tones, the AN model presents a rapid increase in spiking activity at the onset and a gradual decrease afterwards (Figure 2A_1_). The GBC model, to which subthreshold AN inputs converge, shows a “primary-like-with-notch” shape in its peristimulus time histogram (PSTH), which is characterized by a prominent onset peak followed by a short (∼1 ms) pause (Figure 2A_2_). This PSTH shape is regarded as a signature of GBCs (Blackburn & Sachs, 1989; Rhode, 2008; Smith & Rhode, 1987; Spirou et al., 1990). The SBC model, which receives a combination of large endbulb inputs and small bouton inputs, shows a “primary-like” PSTH (Figure 2A_3_) that qualitatively resembles those of AN fibers. The simulated PSTH of the SBC model, however, had a minor second peak (at around 3 ms) associated with refractoriness after the sharp onset response. A similar small dip in response was also found in a number of recordings from putative SBCs (Frisina et al., 1990; Pfeiffer, 1966; Rhode & Smith, 1986; Stasiak et al., 2018; Young et al., 1988). The spontaneous rates were 20.1 (spikes/s) and 98.8 (spikes/s) for GBC and SBC, respectively, matching previous recording results in cats (Smith et al., 1993).

The distributions of first and second spike latencies showed a substantial overlap in the AN (Figure 2A_1_, inset). In GBCs, however, the two distributions were clearly separated from each other (Figure 2A_2_, inset), because the convergence of multiple subthreshold inputs secures the generation of a first spike within a time window of ∼1 ms (Roos & May, 2012; Smith et al., 1993; Young et al., 1988). The spike latency distributions of the SBC model showed an intermediate pattern between AN and GBC (Figure 2A_3_, inset), reflecting the fact that its response pattern is determined primarily by a small number of endbulb inputs from the AN, moderated by the small bouton inputs. Nonetheless, we note that the activation of an endbulb synapse does not always lead to a spike in the SBC model. In our example, the probability of immediate threshold crossing by an endbulb input was 0.64 and 0.51 for the spontaneous and sound-driven activity, respectively. Therefore, 36% of endbulb inputs failed to evoke a spike in SBC at the spontaneous state, and this failure fraction increased to 49% for the sound-driven condition, consistent with previous physiological observations in gerbil SBCs (failure rates of 27 ± 13% for spontaneous activity and 61 ± 19% for sound-driven activity: Nerlich et al., 2014).

In response to low-frequency pure tones, the AN model and BC models showed phase-locked responses with regularly-spaced peaks in their PSTHs (Figure 2B_1_-B_3_). Sharper PSTH peaks for BCs than for AN indicate an enhancement of phase-locking (Joris et al., 1994; Wei et al., 2017, 2023). The simulated spike rates of the three models increased with the sound level at both high (Figure 2C) and low (Figure 2D) frequencies. The increase in GBC spike rate at 350 Hz was more prominent than in AN and SBC rates, suggesting an entrainment of the spiking responses to the stimulus frequency at high sound levels (i.e., spikes occurring at every cycle; Joris et al., 1994). The simulated spike rates of BCs increased with the stimulus frequency up to a few hundred Hz, while the spike rates were roughly constant over 1.5 kHz (Figure 2E). The degree of phase-locking measured with VS first increased with level and then remained stable over 30–40 dB SPL (Figure 2F). At a fixed sound level, an enhancement of phase-locking was observed in both GBC and SBC models, compared to AN (Figure 2G). An increase in VS by adding small synaptic inputs in SBCs was also reported in previous modeling studies (Koert & Kuenzel, 2021; Rothman et al., 1993). These observations, including the enhancement of phase-locking and entrainment in BCs at low frequencies, are consistent with previous physiological observations in cats (Joris et al., 1994), rats (Paolini et al., 2001), gerbils (Typlt et al., 2012; Wei et al., 2017), and chinchillas (Recio-Spinoso, 2012; Wei et al., 2023).

### AM-Tone Responses of BC Models

**Figure 3.**
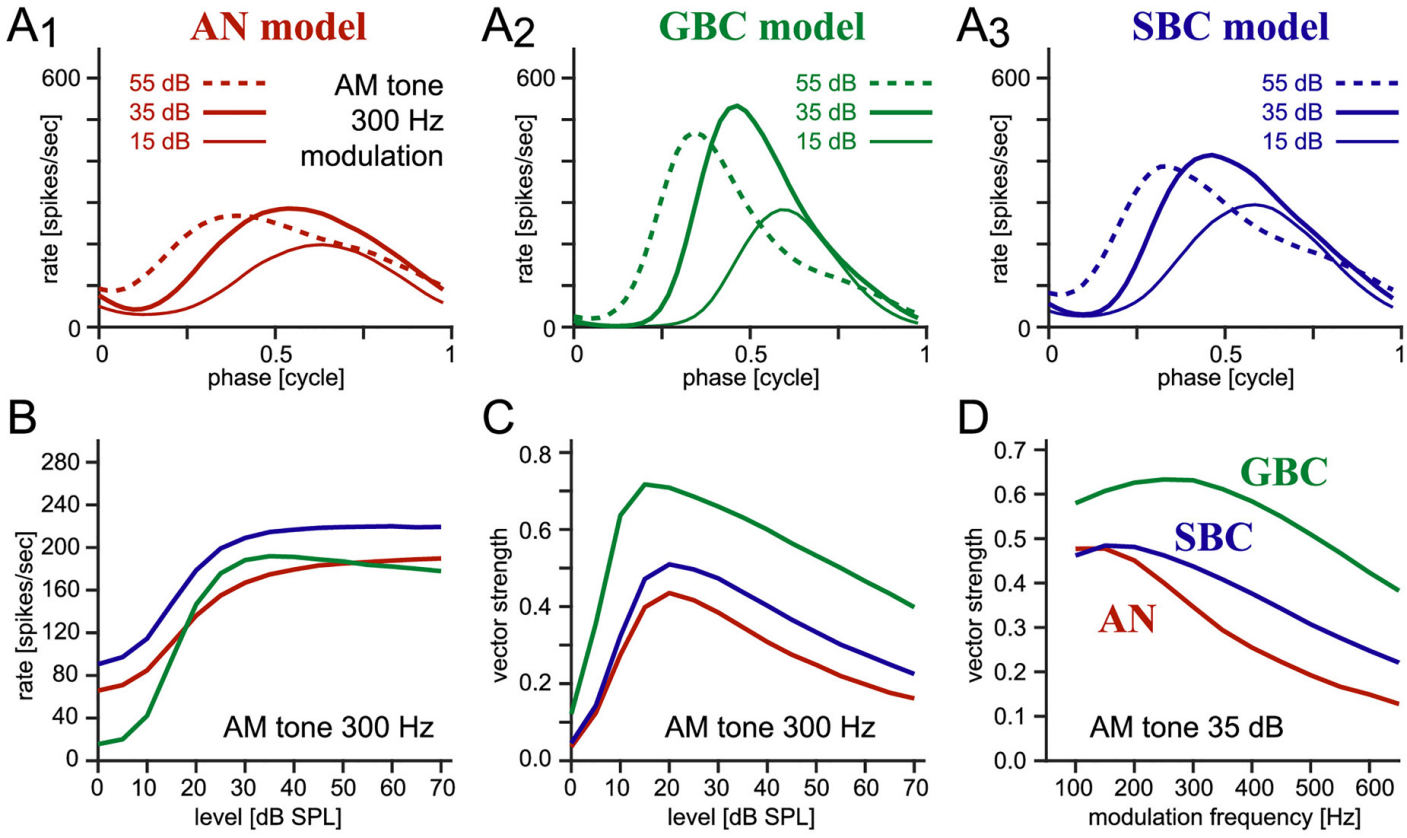
**Simulated responses for am tones. (A_1_–A_3_)** Period histograms of the AN model (red, A_1_), GBC model (green, A_1_), and SBC model (blue, A_3_). The sound stimulus was 7,000 Hz carrier tone modulated at 300 Hz, played at 15 (thin line), 35 (thick line), and 55 (dashed line) dB SPL. **(B)** Rate-level curves for the AN, GBC, and SBC models for 300 Hz AM tones. **(C)** VS-level curves for the three models for the 300-Hz envelope. **(D)** VS-modulation frequency curves at 35 dB SPL.

Next, we explore the spiking activity of BC models in response to AM tones. The CF of the models was fixed to the carrier frequency of 7,000 Hz. The AN model phase-locks to the low-frequency envelope, as shown in its phase histograms (Figure 3A_1_). The peak position of the histogram advances with the sound level (i.e., moving to earlier phases), as in physiological data (Dreyer & Delgutte, 2006; Joris & Yin, 1992). The BC models showed similar peak advancement, but their histogram shapes were steeper (Figure 3A_2_,A_3_) than AN, indicating an enhancement of envelope phase-locking. The simulated phase distributions are slightly asymmetric about the peak, with a steeper increase at earlier phases and shallower decrease at later phases. While the driven spike rate almost monotonically increased with the sound level (Figure 3B), the degree of envelope phase-locking decreased at high sound levels (Figure 3C) for all three models, which is in contrast to pure-tone phase-locking (Figure 2E). Non-monotonic VS-level functions for AM sounds were reported in a number of prior physiological studies (AN: Cooper et al., 1993; Dreyer & Delgutte, 2006; Frisina et al., 1996; Joris & Yin, 1992; Smith & Brachman, 1980; BC: Joris & Yin, 1998; Rhode & Greenberg, 1994a). At a fixed sound level, both BC types showed an enhancement of envelope phase-locking (Figure 3D). The GBC model had more pronounced enhancement than the SBC model, matching experimental observations in cats (Joris & Yin, 1998; Rhode & Greenberg, 1994a) and gerbils (Frisina et al., 1990). Related to the non-monotonic level dependence of phase-locking, low-to-medium sound levels (typically 20–50 dB SPL) were used in previous LSO recordings to examine the temporal coding of binaural AM sounds and broadband noise (Joris, 1996; Joris & Yin, 1995; Tollin & Yin, 2002). We therefore fix the sound intensity level at 35 dB in our following analyses, which is lower than the level used for pure tones. At this level, the spike rates of BCs are almost saturated (Figure 3B) and substantial phase-locking is still observed (Figure 3A_2_,3A_3_,C). Level-dependence of our results will be examined in the last subsection of Results.

### Input Configurations for the LSO Model

**Figure 4.**
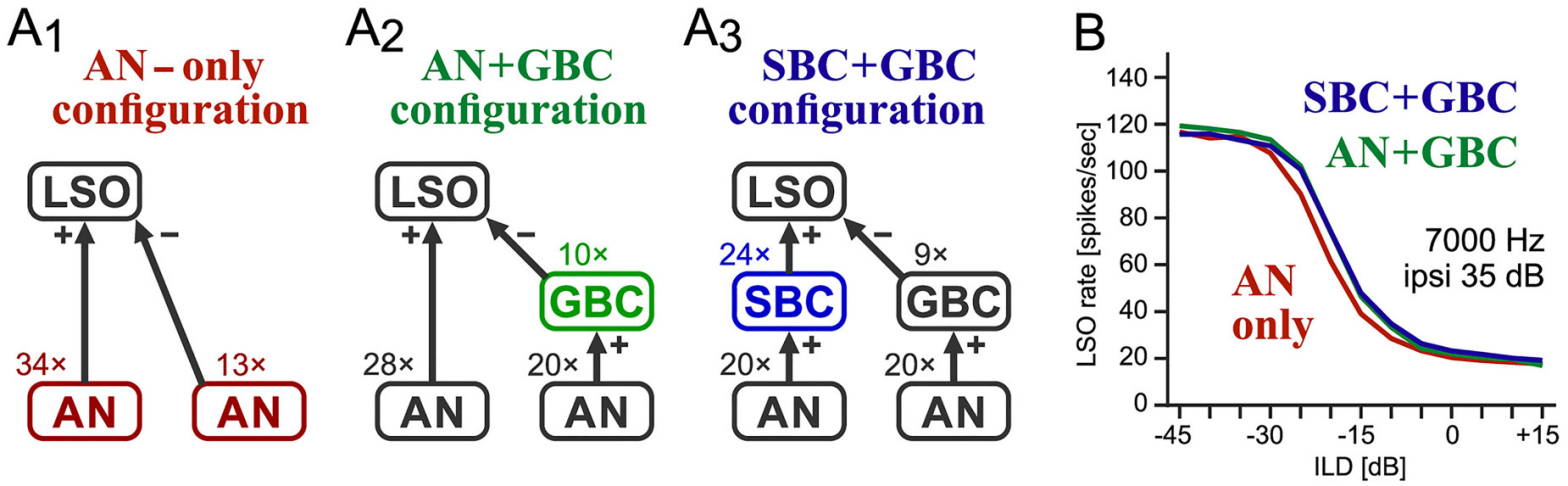
**Simulated LSO circuit. (A_1_–A_3_)** Three different input configurations. The plus and minus signs indicate excitatory and inhibitory projections, respectively. (A_1_) The “AN-only” configuration, in which AN output is directly fed into the LSO model. (A_2_) The “AN + GBC” configuration, in which GBCs are introduced in the contralateral inhibitory pathway while the excitatory inputs are directly provided by AN. (A_3_) The “SBC + GBC” configuration, in which SBCs and GBCs are introduced in the excitatory and inhibitory pathways, respectively. **(B)** Simulated ILD-tuning curves of the LSO model for the three input configurations. The frequency of the binaural tone is 7,000 Hz. The ipsilateral sound level (for excitatory inputs) was fixed to 35 dB SPL, while the contralateral level (for inhibitory inputs) was varied according to ILD.

We have so far examined the biological plausibility of the simulated BC model responses to pure and AM-tones. We now explore how the observed enhancement of phase-locking affects the binaural temporal coding in LSO. To this end, we compare three input configurations (Figure 4A_1_-A_3_). In the “AN-only” configuration, both excitatory and inhibitory inputs to the model LSO neuron are provided directly by the AN fibers on both sides (Figure 4A_1_). This type of input configuration was used also in previous modeling studies (e.g., Brown et al., 2019; Klug et al., 2020). Next, we inserted the GBC stage into the inhibitory part of the circuit (the “AN + GBC” configuration: Figure 4A_2_). The full LSO circuit model, termed the “SBC + GBC” configuration, has BC stages on both sides that receive monaural AN inputs and send excitatory and inhibitory outputs to LSO (Figure 4A_3_). The number of inputs to the LSO neuron was adjusted for each configuration, so that the maximum and minimum spike rates of the ILD-tuning curve for 7,000 Hz tones became 115–120 (spikes/s) and 15–20 (spikes/s), respectively (Figure 4B) (see Ashida et al., 2017 for an explanation of related criteria used in LSO model fitting). Aligning the ILD-tuning curves ensures that all three configurations have comparable excitability for unmodulated sounds, allowing us to compare their coding performances of modulated sounds. In other words, the number of inputs were adjusted so that adding or removing BCs would have minimal effects on ILD tuning. The resulting number of excitatory inputs to LSO ranged from 24 to 34, matching the estimation in the mouse brainstem (Gjoni et al., 2018). The number of inhibitory inputs used was 9–13, which agreed with the measured range in gerbil slices (Sanes, 1990).

### Binaural Temporal Tuning of the LSO Model

**Figure 5.**
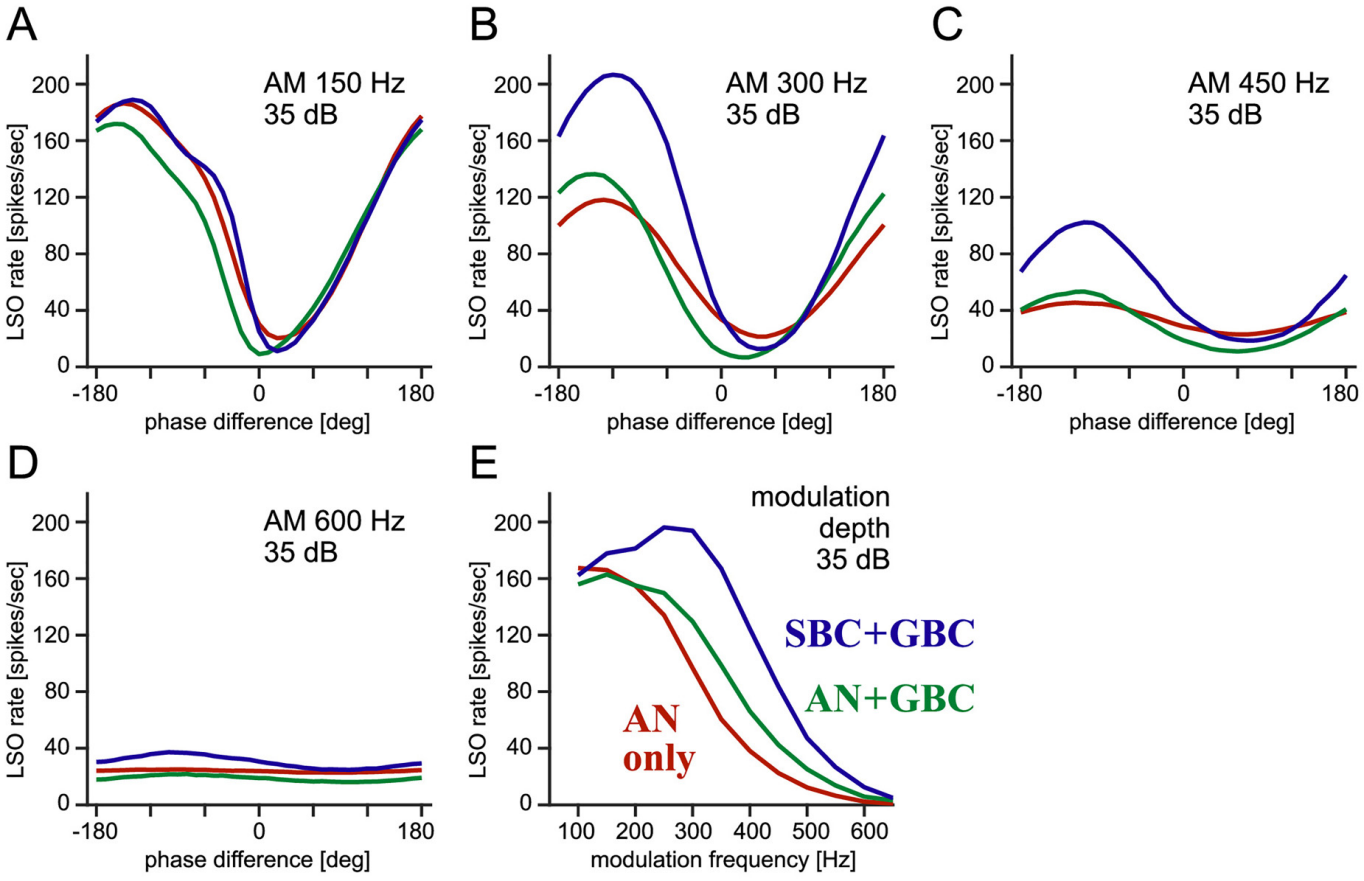
**Simulated binaural envelope-ITD tuning. (A–D)** Simulated phase-tuning curves of the LSO model for the three input configurations. The sound level was fixed to 35 dB SPL, and the envelope frequency was varied (A: 150 Hz, B: 300 Hz, C: 450 Hz, D: 600 Hz). **(E)** Spike-rate modulation depth of the phase-tuning curves (the difference from the peak rate and trough rate) for different modulation frequencies at 35 dB SPL.

After aligning the ILD tunings of the three input configurations, we compare them in envelope-ITD tuning. We presented AM-tones binaurally to the model and examined the output of the LSO neuron by varying the interaural envelope phase differences. At a relative low modulation frequency of 150 Hz, the simulated phase-tuning curves were almost identical between the input configurations (Figure 5A). This indicates that the benefit of BCs is not apparent at this frequency. The simulated tuning curves were slightly asymmetric about the trough, reflecting the asymmetry in phase distributions of AN and BCs (as in Fig 3A_1_–A_3_). Similar asymmetric phase-tuning curves were observed in earlier recordings in cat LSO (Joris & Yin, 1995). For the 300-Hz modulation frequency (Figure 5B), the “AN + GBC” input configuration (green) led to a slightly steeper phase-tuning than the “AN-only” configuration (red). Despite the substantial improvement of phase-locking in GBC at 300 Hz (Figure 3D), the benefit of adding GBCs appeared to be minor here. We speculate that enhanced phase-locking in GBCs would generally lead to a deeper trough in phase-tuning, since they provide inhibitory inputs to LSO; the impact of this sharpening effect, however, may be limited, because the spike rate cannot be below zero. The binaural phase-tuning became substantially sharper by adding the SBC stage to the model (Figure 5B, blue), indicating the contribution of enhanced phase-locking in SBC (Figure 3D). The SBC model had a higher spike rate than AN (Figure 3B), which might also amplify the ITD-dependent response of LSO. To test this possibility, we calculated the total excitatory input rate (average spike rate multiplied by the number of inputs) for the three configurations: AN-only: 5,980 (inputs/s); AN + GBC: 4920 (inputs/s); SBC + GBC: 5,140 (inputs/s). This calculation shows that the total excitatory drive did not increase from the “AN-only” configuration to the “SBC + GBC” configuration, implying that the spike-rate increase at the BC stages was not a major cause for the enhanced ITD-tuning.

At a higher envelope modulation frequency of 450 Hz, the overall activity was reduced for all configurations (Figure 5C), but the advantage of having BCs remained clear (blue). For 600 Hz AM stimuli (Figure 5D), the model with the “AN-only” configuration lost its phase sensitivity (red), while the “SBC + GBC” configuration still showed a small change in spike rate with varied input phase differences (blue). Previous physiological studies also reported that the binaural phase-tuning in LSO diminishes with increasing modulation frequencies (Joris, 1996; Joris & Yin, 1995, 1998). To further test this frequency dependence, we calculated the spike-rate modulation depth (i.e., the difference between the peak and trough rates of a tuning curve) for varied frequencies. This rate modulation depth is a simple measure of binaural tuning performance (e.g., Ashida et al., 2021; Remme et al., 2014). For envelope frequencies below 200 Hz, there was a minimal difference among the three input configurations (Figure 5E). At frequencies over 200 Hz, adding the BC stages substantially increased the rate modulation depth, presenting the benefit of enhanced input synchrony. Moreover, the “SBC + GBC” configuration retained the sensitivity to binaural phase differences up to around 600 Hz. This frequency limit matches previous physiological characterizations of cat LSO neurons recorded in vivo (Joris, 1996).

### Effects of Sound Level on LSO Temporal Tuning

**Figure 6.**
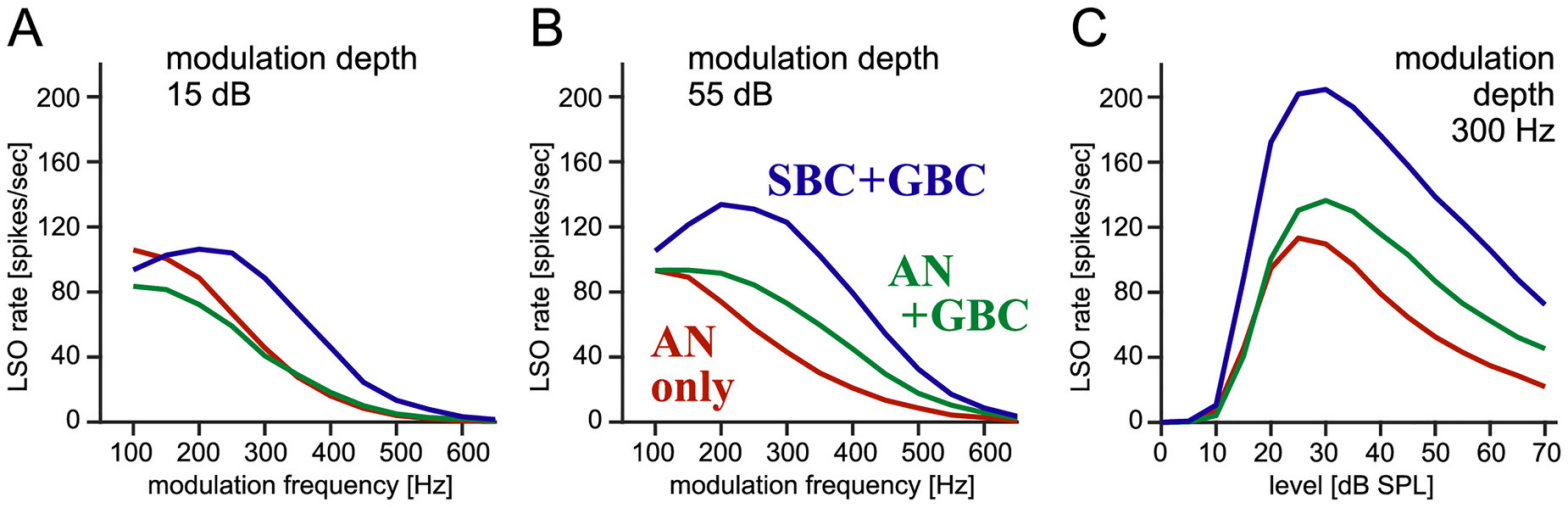
**Effects of sound level on binaural envelope-ITD tuning. (A–B)** Spike-rate modulation depths of the phase-tuning curves for different modulation frequencies at 15 dB SPL (A) and 55 dB SPL (B). **(C)** Spike-rate modulation depth of the phase-tuning curves for 300 Hz AM tones at varied sound intensity levels.

As the final step of our investigation, we examine the benefit of BCs for binaural temporal coding across a range of sound intensity levels. At a low intensity level of 15 dB (Figure 6A), simulated LSO responses showed a higher modulation depth in the “SBC + GBC” configuration (blue) than in the “AN-only” configuration (red) for envelope frequencies of 200 Hz and above. At a higher level of 55 dB, the advantage of including BCs was evident for all tested modulation frequencies (Figure 6B). Compared with 35 dB (Figure 5E), the simulated modulation depths were both smaller at 15 and 55 dB. Two mechanisms likely account for this reduction. First, at intensities below 20 dB, the spike rates of ANs and BCs are considerably lower than their maximal rates (Figure 3B), resulting in diminished overall activity and thus shallower modulation depths of LSO. Second, the degree of envelope phase-locking of ANs and BCs decreases at medium-to-high levels (over 40 dB; Figure 3C), leading to a reduction of LSO modulation depths. Similar level-dependent effects were reported in a study of binaural coincidence detection (van der Heijden et al., 2011). As a result, the modulation depth of simulated LSO responses becomes maximal at 20–40 dB (Figure 6C). Non-monotonic level dependence of envelope-ITD tuning was also observed in physiological recording from the cat LSO (Joris & Yin, 1995). In our simulations, the benefit of BCs for envelope-ITD tuning was consistent across sound intensities from 10 to 70 dB SPL (Figure 6C).

## Discussion

The computation of sound localization cues is one of the most precise temporal information processing in the nervous system (Buonomano, 2007). Various sorts of cellular and synaptic specializations underlie its submillisecond accuracy (reviewed by Keine & Englitz (2025)). The present study developed models of BCs, which project to the binaural nuclei in the auditory brainstem, and demonstrated that an enhancement of temporal synchrony in these cells amplifies the coding performance of binaural envelope-ITD cues in LSO (Figures 5 and 6). Our computational methods complement existing experimental approaches in real animals, by allowing us to explore hypothetical (biologically unrealistic) circuit configurations, in which BCs are fully or partially omitted (Figure 4A_1_–A_2_). Manipulations of input configuration have been shown to be a useful computational approach for the study of binaural processing mechanisms (e.g., Ashida et al., 2021; Dietz et al., 2016).

### Mechanisms of Enhanced Phase-Locking

Various sorts of anatomical and physiological arrangements have been found or proposed to enable the enhancement of phase-locking in BCs. First and probably foremost, coincidence detection of multiple subthreshold inputs generally leads to an increased temporal precision of outputs (Ashida et al., 2019; Kuhlmann et al., 2002; Rothman & Young, 1996; Xu-Friedman & Regehr, 2005). The synaptic structure of GBCs conforms to this hypothesis. In contrast, SBCs receive only a small number of large inputs, making this machinery of enhancement seemingly implausible (Joris & Smith, 2008). Numerous small connections on SBCs were observed histologically (Gómez-Nieto & Rubio, 2009, Ryugo & Sento, 1991), and computational studies demonstrated that a mixed structure of a few large inputs combined with many small inputs can lead to an improvement of phase-locking (Koert & Kuenzel, 2021; Rothman & Young, 1996). The input configuration of our SBC model is founded on this hypothesis. Indeed, our simulated SBC responses showed a better temporal synchrony to low-frequency signals than AN responses (Figures 2F, 3D), in line with previous in vivo physiological observations in animals.

In addition to converging excitatory inputs, anatomical (Campagnola & Manis, 2014; Saint Marie et al., 1991) and physiological (Caspary et al., 1994; Dehmel et al., 2010; Rhode & Greenberg, 1994b) evidence indicate that BCs receive on- and off-CF inhibition from multiple sources. Inhibitory synaptic inputs were shown to boost the temporal fidelity of SBC spikes by suppressing weak, poorly timed synaptic inputs (Keine & Rübsamen, 2015; Nerlich et al., 2014). While our BC models do not explicitly receive inhibitory inputs from non-AN sources, the adaptive threshold partly and implicitly mimics the functions of narrowband, on-CF inhibition. Relevant effects including the inhibition-mediated, activity-dependent changes of spike-generation failures in SBCs (Keine & Rübsamen, 2015) were simulated. To computationally study the effects of broadband, off-CF inhibition, however, a larger-scale simulation would be needed, in which multiple cell types of cochlear nuclei are incorporated (e.g., Manis & Campagnola, 2018). Besides chemical synapses, BCs were found to be connected with neighboring BCs through gap junctions that underlie electrical synapses (Gómez-Nieto & Rubio, 2009, 2011). Local electrical coupling of BCs may contribute to a further improvement of phase-locking through the “first-come-only-served” mechanism (Joris & Smith, 2008), which prefers early, well-timed inputs over delayed inputs.

### Other Types of Binaural Neurons

The mammalian superior olivary complex consists of several monaural and binaural nuclei. The medial superior olive (MSO) is a location where ITDs of low-frequency sounds are computed (Grothe et al., 2010; Yin et al., 2019). Analyses of spike-train correlograms indicated that, compared to the response of AN fibers, the output of BCs has an improved capacity for the binaural coincidence detection of excitatory inputs (van der Heijden et al., 2011). Moreover, a simulation study for the avian homologue of the mammalian MSO demonstrated that the degree of phase-locking roughly linearly affect the performance of ITD-coding (Ashida et al., 2013). We therefore expect that an enhancement of phase-locking in BCs would also lead to an improvement of ITD-tuning in MSO. However, unilateral stimulation of the cochlear nucleus on either side evokes a mixed excitation and inhibition in the mammalian MSO, in which the well-timed inhibitory postsynaptic response precedes the excitatory response (Roberts et al., 2014). A rigorous computational modeling that incorporates the parallel bilateral pathways of excitation and inhibition would be required to verify the contribution of BCs to the ITD-coding in MSO.

The present study focused on the binaural responses of sustained-spiking LSO neurons. Recent experimental studies, however, revealed that LSO is in fact a heterogeneous nucleus in which more than one type of neurons exist (Haragopal & Winters, 2023; Maraslioglu-Sperber et al., 2024). Onset-spiking LSO neurons, which do not (or only weakly) respond to the sustained part of continuous sounds (Franken et al., 2018), are more sensitive and selective to transient sounds (such as clicks) than sustained-spiking neurons (Franken et al., 2021). To simulate the activity of those onset neurons, the LSO model would need to be revised, probably by incorporating an adaptive mechanism (as for our BC models) that rejects continuous, temporally unstructured inputs.

### Heterogeneity of Cells and Synapses

The cochlear nuclei contain anatomically, physiologically, and molecularly distinct neuronal populations; and natural variations are found even within the same neuron type (Cant & Benson, 2003; Jing et al., 2025; Typlt et al., 2012). Endbulb terminals on SBCs were generally regarded as secure synapses, but their failure rate of evoking postsynaptic spikes showed substantial variations among cells (Kopp-Scheinpflug et al., 2002; Nerlich et al., 2014; Stasiak et al., 2018). A population modeling approach, in which model parameters are systematically varied (e.g., Ashida et al., 2019; Goaillard & Marder, 2021), would be needed to cover the experimentally observed response heterogeneity. SBCs in cats are further divided into two subtypes: large SBCs located in the rostral AVCN receiving AN fibers with lower CFs and small SBCs in the caudal AVCN having higher CFs (Cant & Benson, 2003; Osen, 1969). However, this distinction is rather ambiguous in guinea pigs (Hackney et al., 1990). In mice, which lack low-frequency hearing, SBCs are predominantly small subtypes (Cao et al., 2007; Kuenzel, 2019). While their functional differences are still unclear, large and small SBCs may play distinct roles in the sound localization circuits, possibly in a CF-dependent manner (Cant & Benson, 2003). In the present study, the property of the BC models was assumed to be uniform across CFs, leaving the issue of tonotopic variations as a subject of future studies.

The modified endbulb synapses on GBCs also show large variations in size, some of which may be peri- or even suprathreshold (Ostapoff & Morest, 1991; Spirou et al., 2005, 2023). In our GBC model, we simply assumed that all inputs to have the same amplitudes. The effects of input variations on BC outputs are addressed elsewhere (Wang et al., 2026). The postsynaptic response in our BC models have a shared duration of 0.5 ms for the endbulb and modified endbulb synapses. This setting matches the observation in slice recordings, in which the amplitude and time course were measured for spontaneous and evoked synaptic inputs (Cao & Oertel, 2010; Gardner et al., 1999; Isaacson & Walmsley, 1996). As there were no measurements performed for the small bouton inputs on SBCs, we assumed the same response duration for bouton inputs as for the endbulb inputs. It is possible, however, those small inputs have a different time course at the spike-initiation site near the cell body. Particularly for dendritic synapses, membrane filters may delay or broaden the postsynaptic response. A previous multicompartment modeling study reported that the effects of dendritic inputs are stable over a relatively wide range of synaptic time constant (Koert & Kuenzel, 2021).

An early study in cats reported that both SBCs and GBCs are innervated predominantly by high-SR fibers (Liberman, 1991), and therefore we used the high-SR condition for the AN model. There was contradicting evidence, however, for a mixed innervation of high- and low-SR fibers on BCs (Ryugo & Rouiller, 1988). A varied degree of projections from type-Ia (high-SR) and type-Ib/Ic (medium- and low-SR) AN fibers were found to form endbulb synapses (Wang et al., 2021), suggesting functional segregation or gradation in SBC subpopulations. In a recent in vivo recording study in mice, no significant difference was found for the threshold and spontaneous rate of SBCs that receive projections from distinct AN fiber categories (Wong et al., 2025). As medium- and low-SR fibers often present stronger maximum phase-locking than high-SR fibers (Frisina et al., 1996; Joris & Yin, 1992), we expect that the combined projections of all SR fibers would further enhance temporal synchrony in BCs compared to those receiving only high-SR fiber inputs. More detailed experimental characterizations, ideally in multiple species, would be necessary to adequately incorporate and investigate relevant effects of SR in the model (also see the discussion of Spirou et al. (2023)).

### Potential Applications and Required Modifications

The LSO model without a BC stage has been used for previous simulation studies of binaural hearing. Examples include the simulation of binaural interaction components of auditory brainstem responses (Brown et al., 2019), and predicting the behavioral performances of normal-hearing (Klug et al., 2020) or cochlear-implanted (Hu et al., 2023) subjects. By adding the BC stages, biologically more realistic applications would be possible in future studies. We offer, however, some cautionary notes related to the limitations of the model. First, the model parameter set we adopted was not intended to be the “best” parameters in any sense; they were selected from the experimentally measured or estimated ranges, to replicate known physiological responses to a reasonable extent (also see related discussion in Ashida et al. (2017)). Furthermore, as noted above, a single parameter set may be insufficient to replicate the diversity of responses at each stage. Second, incorporating the BC stages would multiply the total computation time by the number of converging AN fibers, as the AN stage is far more computationally demanding than the other stages (Ashida et al., 2019). In our case, the full model (Figure 4A_3_: simulating 660 AN fibers for one LSO) required about 14-fold larger computational costs than the AN-only model (Figure 4A_1_: simulating 47 fibers). Hence, a user of the model should consider whether the added biological details are worth the increase in computational demands. Third, the BC and LSO models in the present study did not consider synaptic and axonal conduction delays and jitters, since our primary focus was on the sustained responses. To closely match the onset response latency to experimental data at each stage (e.g., Roos & May, 2012; Young et al., 1988), artificial spike timing jitters would have to be added (Wang et al., 2026). Such modifications might be important especially for simulations with highly transient stimuli (such as clicks). In natural sounds that modulate both temporally and spectrally, ITDs and ILDs together vary simultaneously (Owrutsky et al., 2021). Accordingly, the response of an LSO neuron to virtual free-field stimuli reflects the changes of both binaural cues (Tollin & Yin, 2002). The model framework presented here may serve as a computational tool to study the distinct roles of AN fibers, SBCs, GBCs, and LSO neurons in a natural acoustic environment.
